# Acute effects of neuromuscular electrical stimulation on lactate, IGF-1 and cognition in chronic spinal cord injury: A pilot randomized cross-over study

**DOI:** 10.1080/10790268.2025.2545067

**Published:** 2025-09-02

**Authors:** Wouter A. J. Vints, Ugnė Mazuronytė, Orgesa Qipo, Oron Levin, Swathi Gujral, Jeanine Verbunt, Charlotte C. M. van Laake-Geelen, Vaida Pokvytytė, Nerijus Masiulis

**Affiliations:** 1 Department of Health Promotion and Rehabilitation, Lithuanian Sports University, Kaunas, Lithuania; 2 Department of Rehabilitation Medicine Research School CAPHRI, Maastricht University, Maastricht, The Netherlands; 3 Centre of Expertise in Rehabilitation and Audiology, Adelante Zorggroep, Hoensbroek, The Netherlands; 4 Frailty in Aging (FRIA) Research Group, Vrije Universiteit Brussel (VUB), Brussels, Belgium; 5 Movement Control & Neuroplasticity Research Group, Group Biomedical Sciences, Catholic University Leuven, Heverlee, Belgium; 6 Department of Psychiatry, University of Pittsburgh School of Medicine, Pittsburgh, PA, SA

**Keywords:** spinal cord injury, Cognitive aging, Neuromuscular electrical stimulation, Dose–response, Myokines, Lactic acid

## Abstract

**Introduction:**

Individuals with spinal cord injuries (SCI) exhibit an accelerated age-related cognitive decline compared to healthy individuals, even after adjusting for mood factors and concomitant traumatic brain injury. We hypothesized that neuromuscular electrical stimulation (NMES) on hamstring and gluteal muscles may induce a dose-dependent increase in lactate and insulin-like growth factor-1 (IGF-1) which is hypothesized to be associated with a temporary enhancement of cognitive performance.

**Methods:**

Twenty-two individuals with chronic SCI participated in a randomized cross-over study, receiving NMES on one of both visits. Participants randomly underwent a single session of 30 or 60-minute NMES. Lactate, IGF-1 levels and processing speed on the Symbol Digit Modalities test (SDMT) were tested before, immediately after and 30 minutes after intervention or 60 minutes rest.

**Results:**

Lactate levels increased significantly immediately after NMES conditions compared to control (*p* = 0.004). Lactate increases were larger in the 30-minute NMES group compared to the 60-minute NMES group, consistent with the higher current amplitude applied in the former (100 mA compared to 40 mA). IGF-1 increases did not significantly differ between groups (*p* = 0.262), and there were no significant differences in SDMT performance changes over time between groups (*p* = 0.892).

**Conclusion:**

Acute NMES did not induce changes in IGF-1 levels or cognitive performance in individuals with SCI. However, 30 min of 100 mA of NMES significantly increased lactate levels, and could be used as a marker of NMES intensity in this population. Further research is required to explore various NMES protocols and their impact on cognitive domains in individuals with SCI.

## Introduction

Cognitive function impairments are present in about 60% of all individuals with a spinal cord injury (SCI) (1,2). Their relative risk for cognitive impairment is 13 times higher than that of the general population (3) and their hazard ratio for Alzheimer’s dementia is twice as high (2,4). Even when excluding individuals with concomitant traumatic brain injury (TBI) and accounting for mood influences, individuals with SCI demonstrate reduced performance in information processing speed, executive function, verbal fluency, short-term memory, long-term memory and attention tasks compared to age-matched healthy adults (5,6). Moreover, these impairments have been reported to worsen over time, indicating an accelerated cognitive aging process (7). Individuals with SCI aged 30–40 years generally exhibit cognitive test results and brain activation patterns during cognitive tasks that closely resemble those of older adult controls aged 50–60 years, while differing significantly from their age-matched counterparts (6,8).

Physical exercise may attenuate the cognitive aging process (9–11). Even a single session of (*i.e.* acute) physical exercise may temporarily enhance cognitive performance (12–14). This effect may partly arise from the release of myokines, factors released into the blood stream from contracting muscle tissue (11,12,15). More than 1,000 putative myokines have been described, some of which may cross the blood–brain barrier and exert neurotrophic effects (11,16). Examples of widely studied myokines are insulin-like growth factor-1 (IGF-1) and brain-derived neurotrophic factor (BDNF), which have repeatedly been reported to influence neuroplastic processes and cognitive performance (11,17,18). During and following an exercise training session, myokines can activate signaling pathways in the brain, facilitating long-term potentiation-like processes that are critical in several cognitive function domains (11,19,20).

However, individuals with SCI may face challenges in engaging in leisure-time physical exercise, depending on the level and completeness of their injury (9). Therefore, neuromuscular electrical stimulation (NMES) is proposed as a promising alternative or adjunct to physical exercise for inducing muscle contractions in this population (21). Moreover, muscle activation with NMES has been shown to induce the release of myokines in healthy individuals (21–24), but no studies have been conducted in individuals with SCI. Additionally, few studies investigated the effect of NMES on cognitive function (21). In one study, 100 stroke patients were allocated either to standard rehabilitation therapy or standard rehabilitation therapy combined with NMES, with electrodes placed above the hyoid bone and below the thyroid notch, for 30 minutes, twice a day, 6 days per week for a total of 4 weeks. The NMES group showed significantly larger improvements on the Mini-Mental State Examination (MMSE) over time (25). In young healthy participants, a single 25-minute session of NMES on the quadriceps was found to increase cardiac output, cerebral blood flow measured with transcranial Doppler ultrasound and cerebral oxygenation measured with functional near-infrared spectroscopy (fNIRS). Moreover, significant increases in memory and executive functioning were noted. The authors argue that the NMES-induced cerebrovascular changes may underly the cognitive performance changes (26), as also reported in physical exercise studies (27). Another study explored the effects of two 30 min sessions of NMES to the quadriceps muscles with one-week intervals in humans and rats, finding enhanced executive functions and reduced anxiety levels in young healthy adults and elevated BDNF levels in rat hippocampus. They argued that their findings point in the direction of a humoral muscle-brain interaction mediated not by muscle-derived or circulating BDNF, but by lactate (28). This is supported by a previous study showing that blocking lactate entry into the brain abolished hippocampal BDNF expression, while lactate administration activated BDNF expression similarly to exercise training (29). To the best of our knowledge, only one other electrical stimulation study exists in persons with SCI. This study showed improvements in a working memory task after 6 months of functional electrical stimulation (FES)-assisted rowing exercise in individuals with SCI (30).

Our primary objective was to investigate if a single session of NMES on gluteal and hamstring muscles of individuals with SCI can induce (a) immediate changes in cognitive performance on the Symbol Digit Modalities Test (SDMT) and (b) immediate changes in IGF-1 and lactate levels. Secondarily, we tested if the NMES-induced effect on lactate and SDMT performance lasted 30 minutes after the intervention. Thirdly, as an indicator of dose–response, participants were randomly allocated to either 30-minute or 60-minute NMES. We hypothesized there would be a dose–response effect causing 60 minutes to induce the largest increase in IGF-1, associated with a larger benefit on cognitive performance.

## Methods

### Participant characteristics

**Figure 1:**
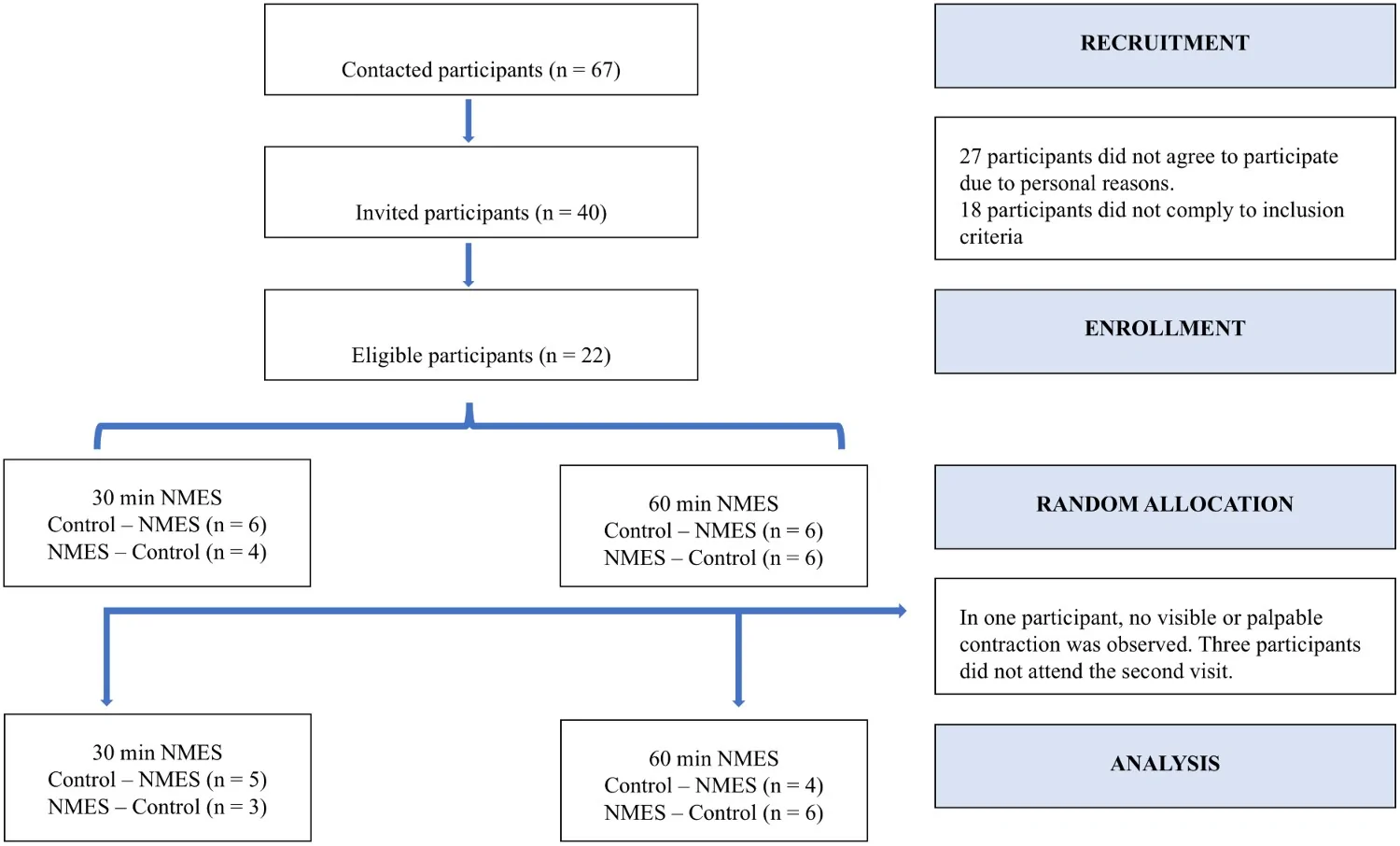
Participant flow diagram.

A total of 67 individuals with SCI were invited for the study, of which 40 agreed to participate. Twenty-two participants met the inclusion criteria. Participants were recruited from local SCI associations and activity clubs in Lithuania (see Fig. 1). Participants were aged between 25 and 54 years, with a chronic traumatic SCI (between 7 and 37 years since injury), injury level L1 or higher and an American spinal cord injury Association (ASIA) Impairment Scale (AIS) classification A to C (31). Reasons for exclusion were the inability to lie in prone position for the duration of the NMES session, flaccid paresis (areflexia) which might indicate lower motor neuron disease, a history of severe autonomic dysreflexia, insufficient mastery of the Lithuanian language, severe cognitive or communicative disorders, intolerance or contra-indication for electrical stimulation (including current cancer, pregnancy, or metal implants in the stimulation area), recent or current participation in an electrical stimulation program (up to 6 months prior to inclusion), recent or current pressure ulcer category 2 or higher, and infections within 3 weeks prior to blood sample collection. Participants were allowed to withdraw from the study at any time. Written informed consent was obtained from all participants or their legal representatives before inclusion in the study. Approval for the study was obtained from the Bioethics Committee of the Lithuanian Sports University Nr. 2021 07 07 NR. BNL  – KIN(B)−2021-402.

### Study protocol

**Figure 2:**
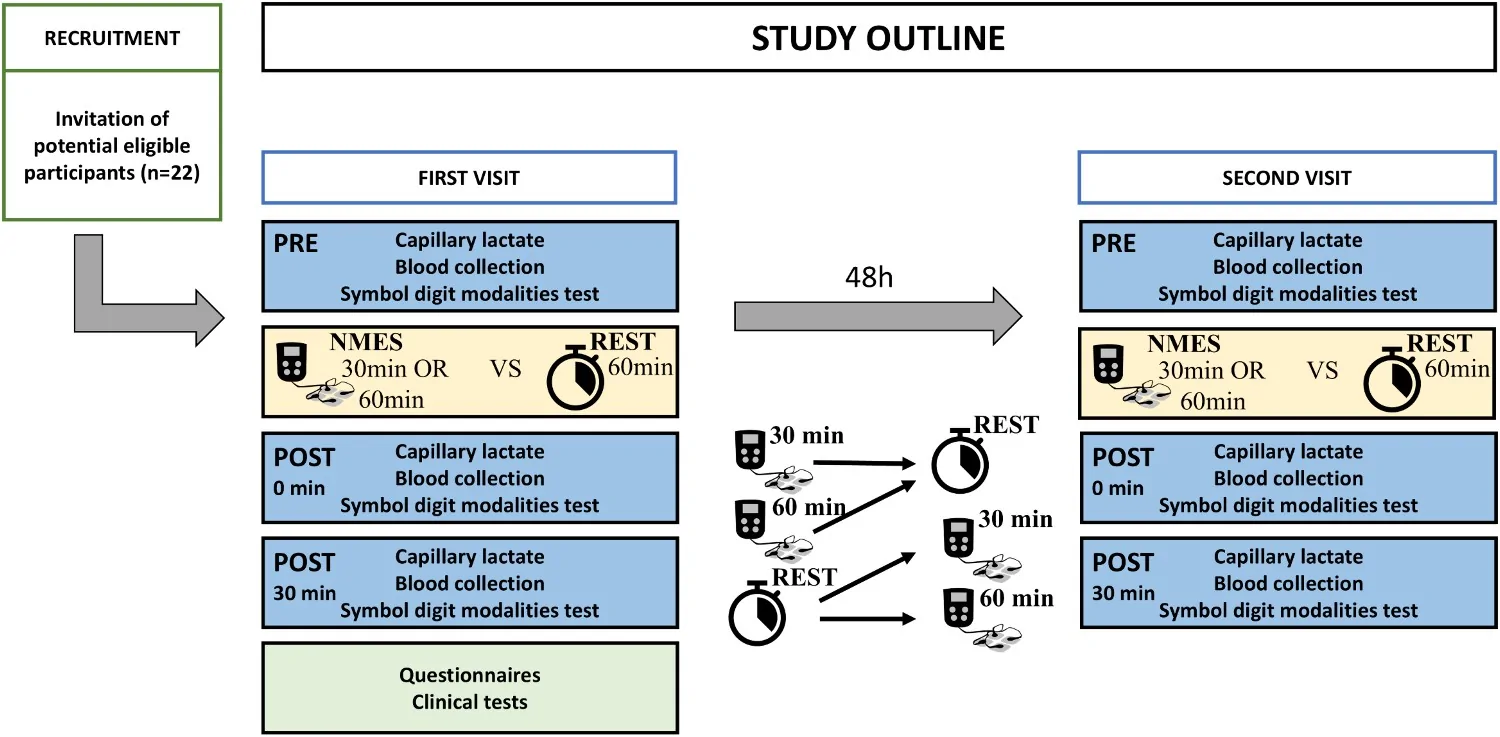
AB/BA/AC/CA cross-over study outline. Abbreviations: NMES, neuromuscular electrical stimulation.

Participants were enrolled in a randomized AB/BA/AC/CA cross-over study. Randomization was done by means of random number calculator in Excel. Participants were randomly allocated to a 30 minutes (n = 10) or 60 minutes (n = 12) experimental NMES condition and randomly determined to either undergo the control condition (60 minutes of rest in waiting room, n = 12) first and the experimental condition second (n = 10), or vice-versa (see Fig. 1). The arrival order of the participants (time of the day measurements took place) was determined randomly by one researcher unaware of the testing day schedule or allocation of the participant. They were invited to visit the Lithuanian Sports University in Kaunas, Lithuania on two separate days with exactly 48 h between both visits. Before the first visit, participants were informed about the study content by the recruiting researcher (author V.P.), and a participant information form, including an informed consent form, was provided electronically. On the first visit, they were first asked to complete the informed consent form before the start of the experiments. Subsequently, participants underwent lactate assessments, blood collection and cognitive assessments in this order. This was followed by 30-minute NMES, 60-minute NMES, or 60 minutes of rest in a waiting room, according to the participant allocation. Immediately after intervention or rest and 30 min later, assessments of lactate, blood collection and cognitive tests were repeated in the same order. On the first visit, participants were asked to complete the questionnaires and undergo the clinical tests. On the second visit, participants did not undergo any additional tests apart from the intervention and outcome measures (see Fig. 2). Assessors were the same between visits. During testing and interventions, participants and assessors were aware of the participants’ group allocation.

### Demographic and clinical characteristics

Participants were asked to provide a medical history file from their general practitioner including a description of chronic illnesses and the level and completeness of spinal cord injury based on an AIS classification (31) measured at least two years after the date of injury. If this information was not available, the AIS-classification was re-evaluated by a trained rehabilitation physician (author W.V.). Furthermore, participants were specifically asked to report a history of traumatic brain injury (concomitant or non-concomitant to the spinal cord injury), diabetes mellitus, oncologic disease, systemic inflammatory disease, infections in the last 3 weeks, wounds in the last 3 weeks, or autonomic dysreflexia. We asked participants to report their age, sex, drug or alcohol abuse, smoking status, time since spinal cord injury, highest educational degree (*i.e.* primary education, secondary education, high school, university bachelor, or university master), current job or indication of unemployment or retirement, and participation in physical exercise (type, sessions per week, duration per session). Finally, we calculated body mass index (BMI) for each participant based on the measured body weight and self-reported body length.

### Health-related questionnaires

Traumatic brain injury (TBI)−4 questionnaire: The probability of concomitant traumatic brain injury at the moment of the SCI was assessed with the TBI-4 questionnaire screening tool (32,33). Participants were asked if they lost consciousness or had a temporary loss of memory at the time of injury. If they indicated that this was not the case, concomitant TBI probability was ranked ‘improbable’. If they did not lose consciousness but reported other mental status changes immediately following SCI, TBI probability was ranked ‘possible’. If they lost consciousness, they were asked to specify the duration of unconsciousness, with a duration below 30 minutes, longer than 30 minutes, or more than 24 hours considered indicative of mild, moderate or severe TBI respectively. It should be noted that the TBI-4 questionnaire has good sensitivity, but poor specificity. Therefore, the results are merely indicative of a possible TBI risk (32,33).

### Lactate assessment

Capillary lactate levels were measured using the Lactate Pro 2 (Arkray Co., Ltd.) from a drop of blood collected on a test strip after a pin-prick on the fingertip. The first drop of blood was discarded and wiped away with a sterile tissue. Lactate measurements were performed before, about 1–2 minutes after, and 30 minutes after the intervention or control condition.

### Blood biomarker assessment

Insulin-like growth factor-1 (IGF-1) was measured from venous blood samples drawn at the antecubital vein before and five minutes after the intervention or control condition. Only IGF-1 levels at baseline and IGF-1 levels immediately after intervention or control were measured. Additionally, we only assessed IGF-1 levels from the 17 participants from whom we had a complete set of blood samples (pre and post on both the first and second visits). The reasons for missing samples included not showing up for the second visit (n = 3), insufficient blood collected for processing (n = 1) and refusal of follow-up blood collection (n = 1). Participants’ initial blood draw occurred at any time of day. On the second study visit, arrival time was kept the same and participants were asked to take the same meal in order to account for diurnal or nutritional influences on blood biomarker levels. Blood samples were collected in serum separator tubes. After blood collection, the tubes were gently inverted 8–10 times and allowed to clot for exactly 30 minutes at room temperature. Then, they were centrifuged for 15 minutes at 4000 x g. After centrifugation, serum was aliquoted into 1.5 mL polypropylene tubes, and subsequently stored at – 80 °C until further analysis. Serum IGF-1 levels were assessed using an enzyme-linked immunosorbent assay (ELISA) from a commercially available ELISA kit (IBL International, GMBH, Germany, MD58011, with a lower limit of detection 0.09 ng/mL). The ELISA measurement was performed by a trained researcher with a background in pharmacology (author O.Q.).

### Cognitive assessment

The symbol digit modalities test (SDMT) (34) was used for cognitive assessment in a quiet room. This test measures processing speed, complex visual tracking and working memory. The oral version of this test has been proven to be reliable in spinal cord injury patients (35). It has been validated for the Lithuanian population, including among multiple sclerosis patients (36). In this test procedure, the subject is given a sheet of paper at the top of which is printed the key (9 abstract symbols and 9 corresponding numbers). The key is available to the participants throughout the test. A sequence of 120 symbols, each printed in a square, is presented below the key. Empty squares are located below the symbol containing squares. In the oral version of this test, the examiner, on a copy of the test sheet, records in the empty squares the numbers the participant associates, orally, with the symbols. In the first test phase, the participant can complete 10 trial symbol-number associations without a time limit; the examiner corrects the participant’s errors. After this familiarization phase, the test begins. Then, participant get 90 s to complete as many associations as possible. The outcome measure is the number of correct associations made in the given time frame. We had three versions of this test available, each with a unique legend containing slightly different symbols. The SDMT test was administered before, about 10 minutes after (immediately after lactate and blood collection), and 40 minutes after the intervention or control condition. The order of the versions used was 1-2-3 for the first visit and 2-1-3 for the second visit.

### NMES intervention

NMES was applied using a battery-powered portable stimulator with surface electrodes (CEFAR REHAB X2, CEFAR Medical AB, Sweden). For each leg, one electrode was placed on the proximal side of the gluteal muscles, and a second electrode was positioned over the mid-part of the hamstrings, while participants lay in prone position on an examination table. NMES induced a tetanic contraction of the gluteal and hamstring muscles. Stimulation on this site was of interest, because it has previously been reported that NMES can possibly reduce the risk of pressure ulcers in people with SCI (37). The downside was that this set-up did not allow us to measure force production, a reason why most NMES studies target the quadriceps muscles. Participants underwent one of the two possible interventions (*i.e.* 30-minute or 60-minute NMES). For both NMES interventions, electrical stimulation involved a biphasic 6-second to 18-second activation-rest cycle, 50 Hz frequency, and a 400 μs pulse duration. The activation within the activation-rest cycle consisted of a 1.5-second ramp-up, a 3-second full activation and 1.5-second ramp-down followed by 18 s of rest. The current amplitude was gradually increased until at least a visible or palpable muscle contraction was achieved. Then, the amplitude was further increased as long as it did not cause discomfort in the participant. However, as some participants with SCI may never start to feel any discomfort upon increasing intensity, it was predetermined that the current amplitude would never be elevated above 40 mA in the 60-minute intervention group, and above 100 mA in the 30-minute intervention group. The reason for this decision was that we expected a risk of muscle fatigue when longer durations would be used at higher intensities. In persons with SCI, unaccustomed to NMES, submaximal stimulation was reported to induce significant muscle fatigue within three minutes, seen as a decrease in produced force (38). In none of the participants a leg movement against gravity was seen.

Participants in the control condition stayed seated in their wheelchairs in the waiting room for 60 minutes between assessments.

### Statistical analysis and sample size calculation

IBM SPSS Statistics 27 (IBM Inc., Chicago, IL, USA) was used to perform all analyses. The graphical presentation of the data and the estimation of effect sizes with 95% confidence intervals was performed with Data Analysis with Bootstrap-coupled ESTimation (DABEST, version 2023.02.14) (39). First, the data was inspected for outliers and normality. Extreme outliers, defined as values lying more than 3× the interquartile range away from the median were excluded. For this reason, we excluded one outlier in lactate results (on the third measurement in control condition), two outliers in IGF-1 results (one on the second measurement in the control condition and one on the second measurement in the 60-minute condition) and one outlier in the SDMT results (on the third measurement in control condition). Normality was defined as a value of kurtosis and skewness between – 2 and +2. Additionally, normality was visually checked using P–P plots and histograms. Data not meeting one of the normality assumptions were log transformed. Homoscedasticity was tested with Levene’s test.

Differences between 30-minute and 60-minute NMES groups at baseline were analyzed using independent t-tests and Chi² tests (or Fisher Exact tests, if the expected count in any of the cells was below 5) for continuous and categorical variable respectively. Within group changes over time were analyzed using dependent samples t-tests. A three-way repeated measures ANOVA was conducted for the evaluation of changes in cognitive, lactate and IGF-1 levels caused by the intervention compared to the control condition, with one between-group factor (30-minute NMES versus 60-minute NMES) and two within-group factors (pretest versus first posttest versus second posttest; and NMES versus control). Finally, an explorative analysis of the moderating role of certain participant characteristics on the change in the outcome measures was performed using Pearson’s correlations for continuous parameters and independent samples t-tests for categorical parameters. No corrections for multiple testing were applied.

The sample size needed to have sufficient power for performing a three-way repeated measures ANOVA and find meaningful differences in our primary outcome, changes in SDMT test performance, was estimated with a Shiny app, using a simulation-based method to estimate power for ANOVA tests (40). As we could not find previous studies testing acute NMES effects on the SDMT test, we based our simulation on the study of Parthimos *et al.* including 60 male semi-professional basketball players who underwent basketball exercise or one hour inactive resting, with measurements of SDMT test before and after the intervention (41). Based on the results of this study we were only able to assess the sample size needed for a two-way ANOVA test with two within factors. Therefore, we estimated the sample size needed for running this test twice, once for the 30-minute and once for the 60-minute NMES group separately. For the simulation, we entered a high expected correlation among within-subjects factors estimated to be 0.9, a standard deviation of 4.47 and mean values in the exercise group pre 64.93 and post 68.76 and in the control group pre 64.77 and post 65.42 based on the abovementioned article (41). The results of the simulation showed that with a total of 18, 9 participants in the 30 minutes and 9 participants in the 60 minutes group, we would have a power of 86.96 to find differences between exercise and control group, a power of 97.56 to find differences between pre  – and post-tests and a power of 80.14 to find significant interaction effects between the two within factors.

## Results

### Participant characteristics

**Figure 3:**
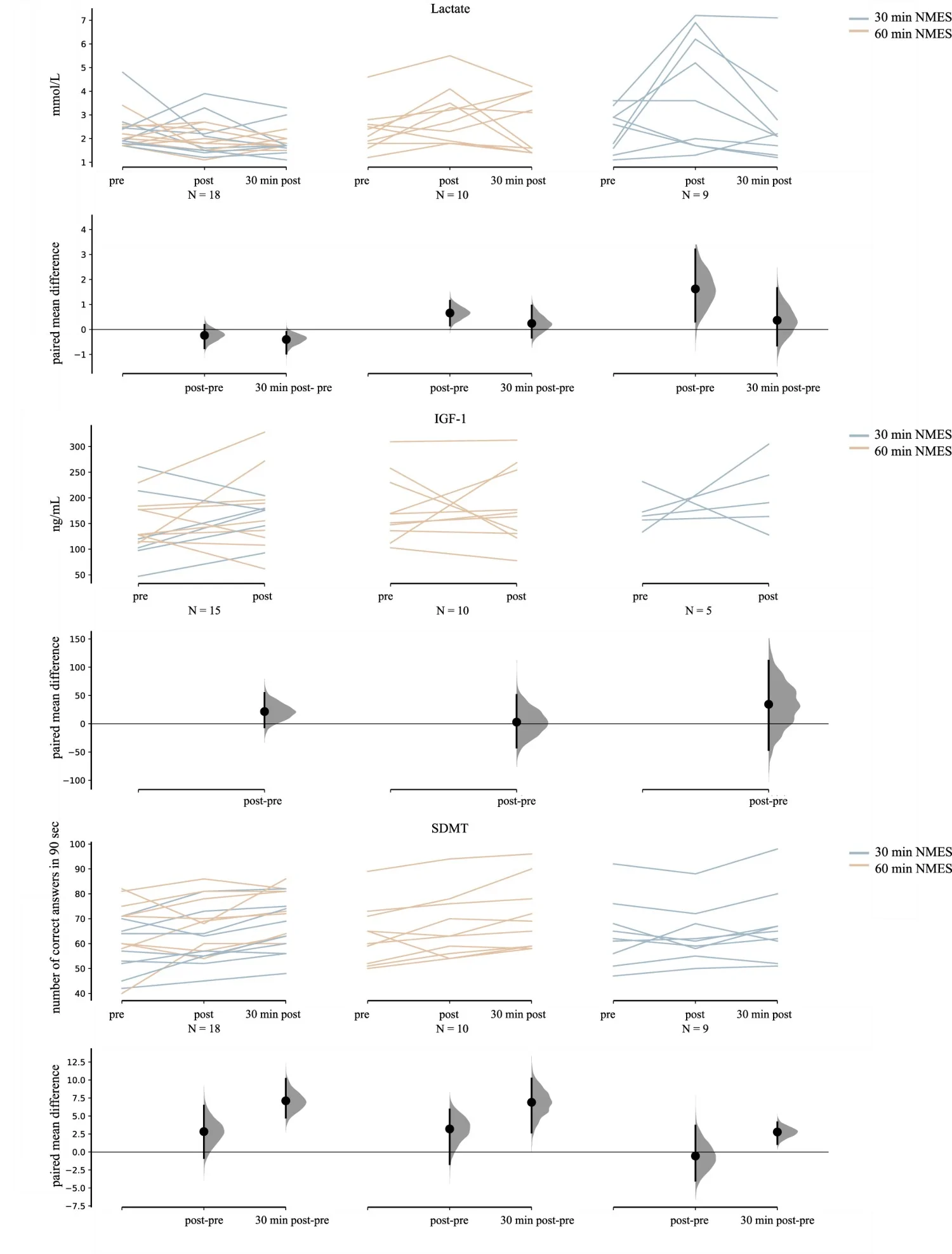
Individual outcome values and effect sizes. The individual values per participant are presented for A) lactate, B) IGF-1, C) SDMT performance over time at baseline (pre), immediately after intervention or control (post) and 30 min after intervention or control (post2), for participants with complete datasets of the respective outcome measure, taking into account missing values and exclusion of outliers. The number (N) of participants with complete data for each condition is shown. Blue lines reflect participants that underwent a 30-minute and orange lines reflect participants that underwent a 60-minute NMES session. Control and experimental conditions are presented separately. The effect sizes of the change per condition and per duration group (paired mean differences) with their 95% confidence interval are presented in comparison with baseline values of the same testing day. Abbreviations: IGF-1, insulin-like growth factor 1; NMES, neuromuscular electrical stimulation; SDMT, Symbol Digit Modalities Test.

Demographic characteristics of the participants are presented in Table 1 and SCI-related characteristics in Table 2. Of the 22 participants included in the study, 19 (86.4%) attended both testing sessions at the Lithuanian Sport University laboratory. The three participants who did not attend the second visit are listed as participants 20–22 (Table 2). Their characteristics or outcome measures did not significantly differ from other participants. Baseline and follow-u*p* values of the individual outcome measures are depicted in Fig. 3. The absolute values of the outcome measures are presented in Table A.1 (supplemental materials). The participants’ ages ranged from 25 to 54 years, and their BMI varied from 15.1–33.5 kg/m². Participants in the 30-minute NMES group were significantly younger than participants allocated to 60-minute NMES group (*p* = 0.019).

**Table 1:** Demographic characteristics.

	60 min NMES (n = 12)	30 min NMES (n = 10)	Total (n = 22)	Significance
Age (years)	43.1 (5.8)	35.8 (7.6)	39.7 (7.5)	0.019*
Sex				NA
Male	8 (66.7%)	10 (100%)	18 (81.8%)	
Female	4 (33.3%)	0 (0%)	4 (18.2%)	
BMI (kg/m²)	24.5 (5.1)	21.7 (5.5)	23.2 (5.3)	0.240
Smoking				0.783
Never	5 (41.7%)	5 (50.0%)	10 (45.5%)	
Past	4 (33.3%)	2 (20.0%)	6 (27.3%)	
Currently	3 (25.0%)	3 (30.0%)	6 (27.3%)	
Alcohol				
Never	2 (16.7%)	3 (30.0%)	5 (22.7%)	0.699
Sporadically	8 (66.7%)	5 (50.0%)	13 (59.1%)	
Regularly	2 (16.7%)	3 (30.0%)	4 (18.2%)	
Education				0.305
Primary education	1 (8.3%)	0 (0%)	1 (4.5%)	
Secondary education	4 (33.3%)	5 (50.0%)	9 (40.9%)	
High school	0 (0%)	1 (10.0%)	1 (4.5%)	
University bachelor	2 (16.7%)	3 (30.0%)	5 (22.7%)	
University master	5 (41.7%)	1 (10.0%)	6 (27.3%)	
Employment				0.840
Not currently	2 (16.7%)	2 (20.0%)	4 (18.2%)	
Currently	10 (83.3%)	8 (80.0%)	18 (81.8%)	
Physical activity				
Sedentary	7 (58.3%)	10 (100%)	17 (77.3%)	NA
Regularly exercising	5 (41.7%)	0 (0%)	5 (22.7%)	
Time since injury (years)	21 (8.6)	14.4 (7.2)	17.9 (8.5)	0.075
Completeness of injury				
AIS A	9 (75%)	8 (80%)	17 (77.3%)	0.594
AIS C	3 (25%)	2 (20%)	5 (22.7%)	
Diabetes mellitus	0 (0%)	0 (0%)	0 (0%)	NA
Reported history of TBI	0 (0%)	3 (30.0%)	3 (13.6%)	NA
TBI-4 questionnaire on concomitant TBI:				
Improbable TBI	5 (41.7%)	6 (60.0%)	11 (50.0%)	0.292
Possible TBI	2 (16.7%)	0 (0%)	2 (9.1%)	
Mild TBI	3 (25.0%)	3 (30.0%)	6 (27.3%)	
Moderate TBI	0 (0%)	1 (10.0%)	1 (4.5%)	
Severe TBI	2 (16.7%)	0 (0%)	2 (9.1%)	

Continuous variables are presented as mean (standard deviation) and unpaired t-tests are used to check for differences between high and low intensity groups (per protocol). Categorical variables are presented as n (%) and Chi² or Fisher’s Exact tests are used to check for differences between high and low intensity groups. If one of the values was 0, a *P*-value could not be calculated, and “NA” was reported instead. Abbreviations: BMI, body mass index; NA, not applicable; NMES, neuromuscular electrical stimulation; TBI, traumatic brain injury

**Table 2:** Individual SCI-related characteristics of included participants.

Number	Level and completeness of injury	Years since injury	Cause	Group
1.	Th10 AIS A	18	Traumatic	Control – 30 min NMES
2.	Th11 AIS A	25	Traumatic	60 min NMES – Control
3.	Th5 AIS A	13	Traumatic	Control – 30 min NMES
4.	Th4 AIS A	13	Traumatic	Control – 60 min NMES
5.	C6 AIS C	22	Traumatic	60 min NMES – Control
6.	C5 AIS A	10	Traumatic	30 min NMES – Control
7.	Th8 AIS A	21	Traumatic	Control – 60 min NMES
8.	Th5 AIS A	7	Traumatic	Control – 60 min NMES
9.	Th8 AIS A	21	Traumatic	60 min NMES – Control
10.	Th12 AIS C	32	Traumatic	60 min NMES – Control
11.	Th4 AIS A	7	Traumatic	30 min NMES – Control
12.	Th10 AIS A	11	Traumatic	Control – 60 min NMES
13.	Th6 AIS C	10	Traumatic	Control – 30 min NMES
14.	C4 ASIA C	23	Traumatic	Control – 60 min NMES
15.	Th12 AIS A	21	Traumatic	Control – 60 min NMES
16.	C5 AIS A	19	Traumatic	Control – 30 min NMES
17.	L1 AIS A	37	Traumatic	60 min NMES – Control
18.	Th6 AIS A	21	Traumatic	Control – 30 min NMES
19.	Th12 AIS A	7	Traumatic	30 min NMES – Control
20.	Th11 AIS A	22	Traumatic	30 min NMES – Control
21.	C5 AIS C	27	Traumatic	Control – 30 min NMES
22.	L1 AIS A	23	Traumatic	60 min NMES – Control

The level of completeness is presented as the AIS score. The time since injury is presented in years. Abbreviations: AIS, ASIA Impairment Scale; ASIA, American Spinal Injury Association.

### NMES effects

Visible or palpable contractions were observed in all participants except one female (participant 7 in Table 2). This participant was allocated to the 60-minute intervention group. She was not included in further analysis. All participants in the 60-minute intervention group received the predetermined maximum intensity of 40 mA. Similarly, all participants in the 30-minute intervention group tolerated the predefined maximum intensity of 100 mA. The ANOVA test results are presented in Table 3. For lactate, the two within-group factors and their interaction were significant (Time, *P* = 0.032; control vs NMES, *P* = 0.036; Time*control vs NMES, *P* = 0.004). Further analysis showed that lactate levels significantly increased with 48% (paired t-test *P* = 0.013) immediately after the experimental conditions (60-minute NMES +25%, 30-minute NMES +67%) while lactate levels decreased with 9% (paired t-test *P* = 0.338) immediately after the control condition, see Table A.1 (supplemental materials). There were no significant findings for IGF-1 changes. Performance on the SDMT only significantly improved over time (*p* < 0.001). No significant effects or interactions were observed for duration of NMES (30-minute vs 60-minute) for any of the three outcome measures (all *p* ≥ 0.259). Notably, performance on the SDMT test tended to decrease immediately after 30-minute NMES (−1%), while it significantly increased in all conditions at 30 min follow-up (Supplementary Table 1).

**Table 3:** Three-way repeated measures ANOVA results.

	Time		Condition (control vs NMES)		Duration (30 vs 60-minute NMES)		Time * Condition		Time * Duration		Condition * Duration		Time * Condition * Duration	
	*p*	_p_η²	*p*	_p_η²	*p*	_p_η²	*p*	_p_η²	*p*	_p_η²	*p*	_p_η²	*p*	_p_η²
Lactate	0.032	0.184	0.036	0.233	0.403	0.041	0.004	0.275	0.371	0.057	0.685	0.010	0.262	0.076
IGF-1	0.231	0.117	0.348	0.074	0.668	0.016	0.808	0.005	0.641	0.019	0.432	0.052	0.259	0.105
SDMT	<0.001	0.451	0.458	0.035	0.337	0.058	0.347	0.061	0.454	0.048	0.464	0.034	0.892	0.004

Dependent variables are presented in the first column. *P*-values and effect sizes (partial eta squared) are given for the Time effect (pre versus post for IGF-1; pre versus immediately post versus 30 min post for lactate and SDMT), condition effect (NMES versus control) and duration effect (30 minutes versus 60 minutes) and their interactions are presented. Significant values are underlined. Abbreviations: IGF-1, insulin-like growth factor 1; *p*, *P*-value; _p_η², partial eta squared value; SDMT, Symbol Digit Modalities Test.

The baseline SDMT results on the second testing day were significantly higher than those on the first testing day, as can be expected due to a practice effect, even when alternate test forms are being used as in this study. The practice effect occurred irrespective of whether the NMES intervention took place during the first (*p* < 0.001) or second (*P* = 0.005) visit. IGF-1 levels were significantly higher at the baseline of the second testing day compared to the first if the first testing day was a control condition (*p* = 0.029).

Pearson’s correlations were used to examine the relationships between age, time since injury, injury level, or BMI related and the changes in outcome measures during the experimental conditions (capillary lactate, serum IGF-1, and SDMT results). Independent samples t-tests were conducted to evaluate how sex, smoking status, alcohol consumption, educational level, participation in physical activity, completeness of injury (AIS A versus C) and loss of consciousness during spinal injury influenced changes in outcome measures from baseline to immediately after NMES (Table A.2 in the supplemental materials). The analyses revealed that changes between outcome measures were not significantly correlated. Participants who regularly consumed alcohol exhibited significantly higher lactate increases (*p* = 0.017) compared to participants who consumed alcohol sporadically or not at all. For participants in the 60-minute NMES group, IGF-1 levels increased significantly less in older individuals (R² = 0.684, *p* = 0.011) and individuals with longer time since injury (R² = 0.524, *p* = 0.042), and SMDT performance increased more in participants with lower injury level (R² = 0.651, *p* = 0.003).

## Discussion

This is the first study to investigate the effects of a single session of NMES on cognitive performance and the role of IGF-1 and lactate level changes in individuals with SCI. Based on data from 18 participants, our findings do not provide evidence of an effect of NMES on IGF-1 level changes or SDMT performance, a measure of processing speed. However, consistent with previous studies (23), our results demonstrated a significant increase in lactate levels following NMES. In able-bodied individuals, lactate level changes were found to be strongly related to the effectiveness of NMES measured as the level of electrically evoked force (42). Previous studies have shown that effectiveness of NMES may vary greatly between individuals due to variability in individuals’ intrinsic tissue properties (43). It can be argued that the variability in intrinsic muscle tissue properties as well as vascularity may be even larger in persons with SCI. As a result, it is unclear if lactate level changes can be used as a surrogate for NMES effectiveness. Given the expected differences in muscle tissue properties between participants, the force produced by a participant with SCI receiving 40 mA could have been higher than that produced by someone receiving 100 mA. However, while lactate levels increased in both NMES conditions in our study, they tended to be larger in the 30-minute NMES group undergoing higher current amplitudes than in the 60-minute NMES group.

Interestingly, although not observed in this study, it has been suggested previously that lactate can also have positive effects on cognition (44). Lactate can serve as a precursor to glutamate and γ-aminobutyric acid (GABA), the main excitatory and inhibitory neurotransmitters in the human brain (45), and potentiate N-methyl-D-aspartate (NMDA) receptors (46). Via these pathways, it was argued that lactate may enhance neuroplastic processes such as long-term synaptic potentiation, potentially boosting learning and memory formation (11,45,46). Furthermore, lactate was described to stimulate the release of BDNF in the blood circulation (23,47) and was shown to mediate hippocampal BDNF mRNA increases in rats (29) and vascular endothelial growth factor (VEGF) in the brain of mice (48), myokines that respectively play a role in neural survival and angiogenesis. One study found higher elevations in BDNF levels following NMES compared to voluntary exercise with the same integrated force of muscle contraction (23). They argued this result was caused by the higher lactate levels that were induced, which is in line with another study showing that lactate levels increased more with NMES than with voluntary muscle contractions (24). Indeed, NMES non-selectively activates both lactate-producing fast-twitch and slow-twitch muscle fibers, while with voluntary exercise first the slow twitch fibers are activated according to the Henneman’s size principle (24).

Despite these mechanisms suggest that NMES might enhance cognition via the release of lactate, our results are not in line with this hypothesis. Given the limited number of studies on this topic (21), we can only speculate as to why our hypothesis was not confirmed. One study described improved cognitive recovery following stroke in persons receiving usual rehabilitation care in combination with NMES with surface electrodes positioned above the hyoid bone and below the thyroid notch compared to usual care. The intervention lasted 4 weeks and cognition was evaluated using the MMSE test (25). In individuals with SCI, we found one other study that evaluated effects of a form of muscle training on cognitive function (30). This study reported improvements in a working memory task after 6 months of FES-assisted rowing exercise. It is unclear whether this effect was partly caused by the FES-assisted exercise intervention or only the result of a practice effect, as there was no control group included in the study (30).

A secondary aim of this study was to evaluate the role of IGF-1 in enhancing cognitive performance in relation to NMES. Physical exercise has been shown to enhance cognitive function, with IGF-1 proposed as one of the potential players underlying this effect (49). Previous studies have shown that intramuscular IGF-1 expression was upregulated after acute NMES in rats (50) and chronic NMES in humans (51). Electrical stimulation in rats even induced a larger intramuscular release of IGF-1 compared to aerobic exercise (50). However, circulating levels of IGF-1 did not significantly increase in the NMES condition compared to control in our study.

It is important to discuss why SDMT performance tended to decrease immediately after the 30-minute NMES session, where participants received stimulation with a higher current amplitude. Maybe this contradiction is in line with physical exercise studies, where it was reported that high-intense physical exercise may either enhance or impair cognitive performance depending on the type of exercise, the type of cognitive task, and the recovery time after exercise and before cognitive testing (13). It is possible that the higher current amplitude in this condition caused discomfort or fatigue, which may have counteracted the typical practice effect observed in cognitive testing. Importantly, none of the participants had autonomic complaints or skin reactions due to NMES. Yet, in some participants the NMES evoked spasticity. Based on these reflections, we advise to use NMES of an current amplitude that is sufficiently high, but still easily tolerated by the participant, similar to what is tolerated by able-bodied individuals.

Second, it is possible that stimulation of paretic muscle tissue may have different effects on the release of myokines and catecholamines than stimulation of healthy muscle tissue. Interestingly, older age and longer time since injury were related to a significantly lower increase in IGF-1 levels following 60-minute stimulation (R = −0.827 and R = −0.724, respectively; Table A.2 in the supplemental materials). Older age was highly correlated to time since injury (R² = 0.664, *P* = 0.001). The age range of participants was relatively large, ranging from 25 to 54 years old and participants in the 60-minute NMES group were significantly older than those in the 30-minute NMES group. The correlation test findings suggest an attenuation of the release of myokines by NMES in case of long-term paresis, possibly associated with larger muscle atrophy.

Third, it may be argued that the cognitive test we used for investigating cognitive changes may not have been the most responsive to changes caused by NMES. A study in persons with SCI reported that an increase in performance of more than 11.3 points in the given 90 s is required before the change from repeated testing can be considered clinically meaningful (35). However, based on knowledge from exercise studies (52,53), we *a priori* expected a good likelihood to find NMES effects on SDMT performance. Firstly, the SDMT is an information processing task with a working memory component, which is known to be one of the domains that is often impaired in people with SCI (5,6). Secondly, meta-analyses investigating cognitive changes induced by a single bout of physical exercise have repeatedly confirmed an enhancement of processing speed and executive functions, which includes working memory (14,52–54). Furthermore, the SDMT test is considered to be of appropriate difficulty, as it has been shown to be crucial to select a cognitive task that is neither too easy nor too challenging, thereby increasing the likelihood of detecting significant effects (55). Finally, we discovered that individuals with higher level of injury improved less on the SDMT test compared to individuals with lower level of injury in the 60-minute group. In line with this finding, Wecht *et al.* (56) found that tetraplegic participants scored lower on a SDMT test compared to paraplegic participants and controls. They attributed this difference to impaired hemodynamic responses in the tetraplegic group, as an inadequate systemic and cerebral hemodynamic response to the SDMT test related to the test score (56). It is possible that hemodynamics also explain our finding that SDMT test changes in participants with higher level SCI are less responsive to an intervention with NMES, as a study indicated that NMES-induced cerebrovascular changes which happen in able-bodied participants such as increases in cardiac output, cerebral blood flow and cerebral oxygenation may be important for cognitive improvements (26). Further research should consider the role of hemodynamics and explore the effect on additional or alternative cognitive tests, specifically related to processing speed, memory or executive functions to expand the knowledge on the effect of NMES on cognitive performance in persons with SCI.

This study has several limitations. First, the study design limited comparability between groups, as the rest period for the control group condition equaled the duration of the 60-minute NMES condition, but not the 30-minute NMES condition. Additionally, different criteria were applied for selecting current amplitudes between the conditions with different NMES duration, which impacted analysis of dose–response effects between the experimental groups. Second, we did not measure the level of electrically-evoked force, a measure that could have allowed us to evaluate the variability in effectiveness among the included participants. Third, it is unclear if the SDMT test applied in this study is sensitive to capturing acute cognitive changes in persons with SCI, while the test is considered a sensitive measure of processing speed typically used in longer trials. For a study presenting clinical meaningful differences in performance in SCI, see Nightingale *et al.* (35). Fourth, it could be argued that measuring additional outcomes might have provided more comprehensive insights from this study, such as including blood assessments of myokines like BDNF, catecholamine levels, measurements of hemodynamics, and evaluating changes across a broader range of cognitive domains using a larger neurocognitive test battery. However, the small sample size of this study and the need to correct *P*-values for multiple testing when adding additional outcomes would likely not have allowed us to perform a valid analysis. Regarding sample size calculation, it should be noted as a limitation that this was based on information from a two-way ANOVA analysis, while the analysis we performed was a more complex three-way ANOVA, which may require larger sample sizes. Additionally, while there is important heterogeneity between persons with SCI included in the study, the study was not sufficiently powered to examine moderators of the effect. This information could be very relevant for further research. The part in which we evaluated the role of between person differences on the outcome measures should only be considered an exploratory analysis, reported in section 3.3 of this manuscript.

## Conclusion

We conclude that a single session of NMES on the gluteal and hamstrings muscles below the level of injury in individuals with SCI induced elevations in lactate levels. On average, this increase was larger in participants receiving higher current amplitudes. We did not find significant changes in IGF-1 levels or SDMT performance induced by a single session of NMES. Further research is needed to determine the role of participant characteristics, the nature of the intervention, or aspects of the outcome assessments with regards to effects on cognitive performance. Future research should also explore the impact of long-term NMES interventions on myokine levels and cognitive function and investigate the role of lactate in relation to other markers of NMES intensity in individuals with SCI.

**Abbreviations**
AIS: ASIA Impairment Scale
ASIA: American Spinal Injury Association
BDNF: brain-derived neurotrophic factor
BMI: body mass index
DABEST: Data Analysis with Bootstrap-coupled ESTimation
ELISA: enzyme-linked immunosorbent assay
FES: functional electrical stimulation
fNIRS: functional near-infrared spectroscopy
GABA: γ-aminobutyric acid
IGF-1: insulin-like growth factor 1
MMSE: Mini-Mental State Examination
NA: information not available
NMDA: N-methyl-D-aspartate
NMES: neuromuscular electrical stimulation
SDMT: Symbol Digit Modalities Test
TBI: traumatic brain injury
VEGF: vascular endothelial growth factor

## Supplementary Material

Appendix A Supplementary table 066.docx
