# Supplementary material for: Acute effects of neuromuscular electrical stimulation on lactate, IGF-1 and cognition in chronic spinal cord injury: A pilot randomized cross-over study
Source: J Spinal Cord Med. 2025 Sep 2;49(5):1095–108. doi: 10.1080/10790268.2025.2545067 (PMC13591980; doi:10.1080/10790268.2025.2545067)
Supplement: Supplementary file 1 [file YSCM_A_2545067_SM2266.docx]

**Appendix A: Supplementary tables**

**Supplementary tables**

| **Table A.1.** Descriptives and changes in outcome measures | | | | | | | |
| --- | --- | --- | --- | --- | --- | --- | --- |
|  |  | Lactate  Mean(SD) Δ(%) | Paired t-test P-value | IGF-1  Mean(SD) Δ(%) | Paired t-test P-value | SDMT  Mean(SD) Δ(%) | Paired t-test P-value |
| Control | PRE | 2.3 (0.8) |  | 152.7 (58.4) |  | 60.8 (12.5) |  |
|  | POST | 2.1 (0.7) -9% | 0.338 | 169.6 (67.3) +11% | 0.202 | 63.4 (11.9) +4% | 0.134 |
|  | POST 2 | 1.9 (0.5) -17% | 0.072 | NA |  | 68.2 (11.5) +12% | <0.001* |
| Experimental | PRE | 2.3 (0.9) |  | 177.0 (55.0) |  | 63.8 (12.4) |  |
|  | POST | 3.4 (1.9) +48% | 0.013* | 189.6 (71.1) +7% | 0.556 | 65.3 (11.9) +2% | 0.339 |
|  | POST 2 | 2.7 (1.5) +17% | 0.386 | NA |  | 68.8 (13.8) +8% | <0.001* |
| 60 minutes | PRE | 2.4 (0.9) |  | 187.7 (65.8) |  | 63.5 (12.0) |  |
|  | POST | 3.0 (1.1) +25% | 0.033* | 200.8 (81.3) +7% | 0.738 | 66.7 (12.9) +5% | 0.127 |
|  | POST 2 | 2.6 (1.2) +8% | 0.506 | NA |  | 70.4 (13.7) +11% | 0.007* |
| 30 minutes | PRE | 2.2 (0.9) |  | 163.4 (37.6) |  | 64.2 (13.6) |  |
|  | POST | 4.0 (2.6) +67% | 0.077 | 176.9 (61.2) +8% | 0.574 | 63.7 (11.2) -1% | 0.800 |
|  | POST 2 | 2.8 (2.0) +17% | 0.573 | NA |  | 67.0 (14.5) +4% | 0.008* |
| Baseline values (PRE), values immediately after control or intervention (POST) and values 30 min after control or intervention (POST 2) are presented as mean (standard deviation). Additionally, the percentage difference compared to baseline values is presented. * P-value < 0.05. Abbreviation: IGF-1, insulin-like growth factor-1; NA, data not available; SDMT, Symbol Digit Modalities Test. | | | | | | | |

| **Table A.2.** Exploratory analysis of the role of participant characteristics | | | | | | | | |
| --- | --- | --- | --- | --- | --- | --- | --- | --- |
|  | Experimental visit change in outcome measures | | | | | | | |
|  | Δ Lactate | | Δ IGF-1 | | | Δ SDMT | | |
| Δ Lactate | R = 1.000 | | R = 0.157  P = 0.736 | | | R = -0.098  P = 0.802 | | |
| Δ IGF-1 | R = 0.157  P = 0.736 | | R = 1.000 | | | R = 0.095  P = 0.839 | | |
| Δ SDMT | R = -0.098  P = 0.802 | | R = 0.095  P = 0.839 | | | R = 1.000 | | |
| Age | R = -0.074  P = 0.763 | | R = -0.212  P = 0.449 | | | R = 0.220  P = 0.366 | | |
| Time since injury | R = -0.223  P = 0.359 | | R = -0.421  P = 0.118 | | | R = 0.251  P = 0.301 | | |
| Injury level | R = -0.279  P = 0.248 | | R = -0.458  P = 0.086 | | | R = 0.337  P = 0.159 | | |
| BMI | R = -0.185  P = 0.449 | | R = -0.297  P = 0.282 | | | R = 0.224  P = 0.356 | | |
| Sex | P = 0.362 | | P = 0.638 | | | P = 0.059 | | |
| Never smoked vs current smoker | P = 0.720 | | P = 0.770 | | | P = 0.615 | | |
| Never/sporadic alcohol consumption vs regularly | P = 0.017* | | P = 0.835 | | | P = 0.141 | | |
| Primary/secondary education versus high school/university | P = 0.807 | | P = 0.665 | | | P = 0.540 | | |
| Employed (yes versus no) | P = 0.489 | | P = 0.965 | | | P = 0.474 | | |
| Physical activity | P = 0.612 | | P = 0.981 | | | P = 0.978 | | |
| Completeness of injury AIS A vs C | P = 0.639 | | P = 0.448 | | | P = 0.168 | | |
| Loss of consciousness | P = 0.998 | | P = 0.798 | | | P = 0.882 | | |
|  | 60-minute NMES | | | | 30-minute NMES | | | |
|  | Δ Lactate | Δ IGF-1 | | Δ SDMT | Δ Lactate | | Δ IGF-1 | Δ SDMT |
| Age | R = -0.155  P = 0.649 | R = -0.827*  P = 0.011 | | R = 0.323  P = 0.332 | R = 0.125  P = 0.748 | | R = 0.666  P = 0.102 | R = -0.154  P = 0.693 |
| Time since injury | R = -0.061  P = 0.859 | R = -0.724*  P = 0.042 | | R = 0.143  P = 0.675 | R = -0.174  P = 0.654 | | R = 0.296  P = 0.519 | R = 0.090  P = 0.817 |
| Injury level | R = 0.379  P = 0.250 | R = 0.684  P = 0.061 | | R = -0.807*  P = 0.003 | R = 0.251  P = 0.515 | | R = -0.061  P = 0.896 | R = 0.360  P = 0.342 |
| BMI | R = 0.303  P = 0.366 | R = -0.468  P = 0.242 | | R = 0.054  P = 0.874 | R = -0.266  P = 0.489 | | R = -0.026  P = 0.956 | R = 0.355  P = 0.348 |
| Sex | P = 0.203 | P = 0.450 | | P = 0.393 | only men | | only men | only men |
| Never smoked vs current smoker | P = 0.375 | P = 0.919 | | P = 0.870 | P = 0.495 | | P = 0.712 | P = 0.611 |
| Never/sporadic alcohol consumption vs regularly | P = 0.127 | P = 0.846 | | P = 0.805 | P = 0.049* | | P = 0.339 | P = 0.106 |
| Primary/secondary education versus high school/university | P = 0.423 | P = 0.845 | | P = 0.938 | P = 0.590 | | P = 0.749 | P = 0.263 |
| Employed (yes versus no) | P = 0.080 | P = 0.967 | | P = 0.914 | P = 0.081 | | P = 0.846 | P = 0.279 |
| Physical activity | P = 0.890 | P = 0.982 | | P = 0.312 | only sedentary | | only sedentary | only sedentary |
| Completeness of injury AIS A vs C | P = 0.115 | P = 0.953 | | P = 0.137 | P = 0.786 | | P = 0.037* | P = 0.601 |
| Loss of consciousness | P = 0.302 | P = 0.968 | | P = 0.938 | P = 0.937 | | P = 0.662 | P = 0.941 |
| The change in outcome measure (Δ) during the experimental condition from before to immediately after NMES was related to several predictors for participants who underwent 30 minutes or 60 minutes NMES. The role of continuous predictors was examined using Pearson’s correlation. The role of categorical predictors was examined using independent sample t-tests. | | | | | | | | |
